# Morphological adaptation of sheep’s rumen epithelium to high-grain diet entails alteration in the expression of genes involved in cell cycle regulation, cell proliferation and apoptosis

**DOI:** 10.1186/s40104-018-0247-z

**Published:** 2018-04-16

**Authors:** Lei Xu, Yue Wang, Junhua Liu, Weiyun Zhu, Shengyong Mao

**Affiliations:** Department of Animal Science and Technology, Jiangsu Key Laboratory of Gastrointestinal Nutrition and Animal Health, Laboratory of Gastrointestinal Microbiology, Nanjing Agricultural University, Nanjing, Jiangsu 210095 China

**Keywords:** Adaptation, Apoptosis, Cell cycle, Proliferation, Rumen papillae, Sheep

## Abstract

**Background:**

The objectives of this study were to characterize changes in the relative mRNA expression of candidate genes and proteins involved in cell cycle regulation, cell proliferation and apoptosis in the ruminal epithelium (RE) of sheep during high-grain (HG) diet adaptation.

**Results:**

Twenty sheep were assigned to four groups with five animals each. These animals were assigned to different periods of HG diet (containing 40% forage and 60% concentrate mix) feeding. The HG groups received an HG diet for 7 (G7, *n* = 5), 14 (G14, *n* = 5) and 28 d (G28, *n* = 5), respectively. In contrast, the control group (CON, *n* = 5) was fed the forage-based diet for 28 d. The results showed that HG feeding linearly decreased (*P* <  0.001) the ruminal pH, and increased the concentrations of ruminal total volatile fatty acid (linear, *P* = 0.001), butyrate (linear, *P* <  0.001), valerate (quadratic *P* = 0.029) and the level of IGF-1 (quadratic, *P* = 0.043) in plasma. The length (quadratic, *P* = 0.004), width (cubic, *P* = 0.015) and surface of the ruminal papillae (linear, *P* = 0.003) were all enlarged after 14 d of HG diet feeding. HG feeding cubically increased the number of cell layers forming the stratum corneum (SC, *P* <  0.001) and the thickness of the SC (*P* <  0.001) and stratum basale (*P* <  0.001). The proportion of basal layer cells in the RE decreased (linear, *P* <  0.001) in the G0/G1-phase, but it increased linearly (*P* = 0.006) in the S-phase and cubically (*P* = 0.004) in the G2/M-phases. The proportion of apoptosis cells in G7, G14 and G28 was reduced compared to the CON (quadratic, *P* < 0.001). HG diet feeding linearly decreased the mRNA expression of *Cyclin E1* (*P* = 0.021) and *CDK-2* (*P* = 0.001) and (*P* = 0.027) the protein expression of Cyclin E1. Feeding an HG diet linearly increased the mRNA expression of genes *IGFBP-2* (*P* = 0.034) and *IGFBP 5* (*P* < 0.009), while linearly decreasing (*P* < 0.001) the *IGFBP 3* expression. The expression of cell apoptosis gene *Caspase 8* decreased (quadratic, *P* = 0.012), while *Bad* mRNA expression tended to decrease (cubic, *P* = 0.053) after HG feeding.

**Conclusions:**

These results demonstrated sequential changes in rumen papillae size, cell cycle regulation and the genes involved in proliferation and apoptosis as time elapsed in feeding a high-grain diet to sheep.

**Electronic supplementary material:**

The online version of this article (10.1186/s40104-018-0247-z) contains supplementary material, which is available to authorized users.

## Background

In modern ruminant agriculture, diets rich in rapidly fermentable carbohydrates are typically used to increase the energy intake and efficiency of meat as well as milk production [1]. However, increased volatile fatty acid (VFA) production and reduced ruminal pH impose a challenge to the absorption and metabolism of the ruminal epithelium (RE) [2, 3]. The RE responds to these challenges in a coordinated manner. Previous studies revealed that RE adaptation to highly fermentable diets entails morphological adaptations associated with tissue proliferation [4, 5]. RE tissue proliferation is presumably a response that maximizes the absorptive surface area for nutrient absorption [6, 7]. Nevertheless, previous studies have largely focused on RE proliferation in calves and dairy cattle during the periparturient period [8, 9]. There is a paucity of data regarding RE proliferation in feedlot ruminants in spite of the fact that dietary transition from backgrounding diets to finishing diets should elicit such a response [8].

RE proliferation is well known to be a response that maximizes the absorptive surface area for nutrient absorption [10–12]. Dirksen et al. [10] showed that a maximal increase in ruminal papillae surface area required six to eight weeks after an increase in the dietary energy density. Bannink et al. [13] found that maximal surface area required approximately three to four weeks after parturition in postpartum cows fed rapidly increased dietary concentrate. However, Reynolds et al. [14] reported that there was no difference in rumen papillae surface area between the prepartum and postpartum phases in Holstein cows fed diets based on NRC (1989) recommendations. Therefore, the results of studying the impact of time on maximizing the absorptive surface area for nutrient absorption during concentrate adaptation were inconsistent [10, 13, 14]. A clear understanding of the time period is required, which might allow for better management of the rumen ecosystem and the practical use of these results in feedlot ruminant production systems.

The surface area enlargement of ruminal papillae during high-grain feeding might attribute to an increased rate of gene transcription related to epithelial proliferation [8] and cell apoptosis [15]. A recent report showed that an increase in dietary concentrate from 10 to 35% caused an accelerated ruminal cell cycle and promoted ruminal cell apoptosis. Short volatile fatty acid and acidic pH may modulate the genes involved in this process [15]. However, to date, little is known about the molecular basis of epithelial proliferation and apoptosis in the RE cells of sheep during grain adaptation. The changes in their cell cycle have not been clarified yet.

In the present study, we hypothesized that the morphological adaptation of sheep’s rumen epithelium to a high grain-diet entails alteration in the expression of genes involved in cell cycle regulation, cell proliferation and apoptosis. Thus, the objectives of the study were to characterize changes in the relative mRNA expression of candidate genes and proteins involved in cell cycle regulation, cell proliferation and apoptosis in the RE of sheep during grain adaptation, and the time period required to maximize the absorptive surface area for nutrient absorption during adaptation was also evaluated.

## Methods

### Animals, experimental design, and diets

All experimental designs and procedures for this study were approved by the Animal Care and Use Committee of Nanjing Agricultural University (SYXK(Su)2015–0656), following the requirements of the Regulations for the Administration of Affairs Concerning Experimental Animals (The State Science and Technology Commission of P. R. China, 1988).

In this study, 20 male sheep (Hu Sheep, BW of 25.60 ± 0.41 kg, mean ± SD, approximately 180 d of age) were placed in individual pens (1.2 m × 1.4 m). These sheep were divided into 4 groups based on age and body weight (BW). At the beginning of the study, all sheep were fed a forage-based diet containing 96.4% hay and 3.6% of a mineral and vitamin premix (the nutrient compositions of the hay are presented in Table 1) on a dry matter (DM) basis for at least 4 wk. In the adaptation stage following the preparation period, three high-grain (HG) diet groups were fed a step-up diet (HG diet was increased 15 by 3.50% of BW per day gradually) for 4 d until fed by 60% concentrate mix (Table 1); the control group (CON, *n* = 5) continued to be fed the forage-based diet. Following adaptation, CON was fed the forage-based diet for an additional 28 d. In contrast, the HG groups received an HG diet for 7 (G7, *n* = 5), 14 (G14, *n* = 5) and 28 d (G28, *n* = 5), respectively. The diets were fed twice in equal portions at 0830 and 1630 h daily. The BW of sheep was measured on the first day of every week before feeding. Sheep in CON were slaughtered after 28 d of hay feeding and sheep in the HG7, HG14, and HG28 groups were slaughtered after 7, 14, and 28 d of HG feeding, respectively. During the experiment, the sheep had free access to clean drinking water, and the BW of sheep were measured on the first day of every week before morning feeding. The health and feed intake of the animals were constantly monitored. Samples of the feed were offered, and the residue removed were collected and weighted daily, and then analyzed for dry matter intake (DMI).

**Table 1:** Ingredient and chemical composition of the diet (DM basis)

Item	Diet	
	Hay	High-grain diet
Ingredient composition, % DM		
Oat hay	63.40	26.00
Alfalfa hay	33.00	14.00
Corn meal	0	34.20
Wheat meal	0	18.00
Soybean meal	0	4.20
CaCO_3_	1.00	1.00
NaCl	0.40	0.40
CaHPO_4_	1.20	1.20
Mineral and vitamin supplement^a^	1.00	1.00
Nutrient composition^b^		
DE, MJ/kg DM	8.88	11.73
CP, % DM	11.18	11.92
Crude fat, % DM	2.09	2.49
Crude fiber, % DM	28.41	12.90
NDF,% DM	44.45	24.54
ADF, % DM	19.52	10.15
Crude ash, % DM	8.34	4.53
Starch, % DM	3.25	32.34

^a^Contained 16% calcium carbonate; 102 g/kg of Zn; 47 g/kg of Mn; 26 g/kg of Cu; 1,140 mg/kg of I; 500 mg/kg of Se; 340 mg/kg of Co; 17,167,380 IU/kg of vitamin A; 858,370 IU/kg of vitamin D; and 23,605 IU/kg of vitamin E

^b^Values were analyzed based on the Feed Database in China [60]

Sheep assigned to CON (*n* = 5), G7 (*n* = 5), G14 (*n* = 5) and G28 (*n* = 5) received a high-grain diet for 0, 7, 14 and 28 d, respectively

### Sample collection

Feed ingredients were sampled at the beginning and end of the experimental period. At the end of each feeding period, the sheep were slaughtered for sampling (4 h after the last feeding) in a local slaughterhouse. Blood samples (5 mL) were taken from the jugular vein immediately before slaughter. Blood was collected using a blood collection tube containing 40 kIU Na-heparin/mL blood. Plasma was harvested by centrifuging the blood samples at 2,000×*g* for 10 min at 4 °C and subsequently storing them at − 20 °C until IGF-1 concentration analysis. Sheep were stunned by captive bolt and killed by exsanguinations according to the animal protection laws of China. Immediately after slaughter, the pH value was determined from a representative sample of rumen fluid (at least 200 mL) by a portable pH meter (PB-10; Sartorius, Goettingen, Germany). Then the rumen fluid was filtrated through four layers of cheesecloth preserved in 25% (wt/vol) metaphosphoric acid and stored at − 20 °C until later VFA concentration was determined using a capillary column gas chromatography (GC-14B; Shimadzu, Japan; Capillary Column: 30 m × 0.32 mm × 0.25 mm film thickness; Column temperature = 130 °C, injector temperature = 180 °C, detector temperature = 180 °C) [16]. Within 5 min of slaughter, rumen tissues from the ventral sac were collected, and the muscular and serosal layers were peeled off by blunt dissection. Serosal layers were transferred into liquid nitrogen and immediately stored at − 80 °C until being analyzed for mRNA and proteins. Other parts of rumen epithelium samples from the ventral sac were isolated, immediately rinsed, soaked in buffer solution and transferred to the laboratory for cell cycle and apoptosis analysis. Rumen tissue (1 cm^2^) from the rumen ventral sac was fixed in 4% neutral paraformaldehyde solution for histomorphometric microscopy.

### Plasma IGF-1 concentration

The concentration of IGF-1 in blood plasma was determined by an IGF-1 RIA KIT (Nanjing Jian Cheng institute of Bio-engineering, Nanjing, China) with a minimum detection limit of 0.1μg/mL at Nanjing General Hospital. Pretreated plasma samples were diluted until their IGF-1 concentrations were in the range of 25 to 100 μg/mL, relative to the reference IGF-1.

### Determination of rumen papillae morphology

Tissue samples from the rumen ventral sac of all sheep were prepared for light microscopy histomorphometric analysis using the methods previously described by Odongo et al. [6]. Samples were fixed in 4% paraformaldehyde, dehydrated, cleared and embedded in paraffin (3 paraffin blocks per animal). Five discontinuous slices per paraffin block per animal were sectioned at a 6 μm thickness, stained with hematoxylin and eosin, and mounted for analysis. The standard sectioning procedure was performed exactly as described by Holle and Birtles [17]. The microscopist was blinded to treatment conditions during the histomorphometric analysis. Five rumen papillae per slide were selected at random for analysis. The length and width of papillae were measured using Image Pro Plus software (Media Cybernetics, Bethesda, MD, USA). The density of papillae (1 cm × 1 cm) was determined using a magnifying mirror (MG3B-1A, Shanghai, China). The total surface of papillae/cm^2^ was calculated as length × width × 2, multiplied by the density of papillae (number of papillae/cm^2^). Measurements of each stratum were made using the 40× objective lens, and five images (five different areas in one rumen papillae) were captured per papillae for a total of 25 replicates per slide per animal. Image Pro Plus software (Media Cybernetics, Bethesda, MD, USA) was used to measure predefined criteria previously described by Steele et al. [5]. In short, the stratum corneum (SC) was the outermost cell layer, which was heavily stained, and the stratum granulosum (SG) was defined as the layer of long axes cells that lay perpendicular to the stratum spinosum (SS) and basale (SB). SS and SB were cells nested between the lamina propria and SG (Additional file 1: Figure S1).

### Cell cycle and apoptosis analysis by flow cytometry

RE tissue (10 g of wet weight, sampled from the rumen ventral blind sac) was digested by 0.25% trypsin and added to 0.02% EDTA in D-Hanks’ solution. This method was described previously [15]. Briefly, the RE from the ventral blind sac of each sheep was quickly excised, then transferred into ice-cold PBS (pH 7.4) and placed in an electric-heated thermostatic water bath at 37 °C, with continuous stirring. The digestion solution was changed every 30 min until the cells were completely dissociated. Cells were washed in a culture medium. A portion of the isolated cells was fixed in 75% ethanol, washed and treated with PBS, followed by 30 min of propidium iodide incubation. Other isolated cells were incubated for 30 min with Annexin V-FITC/PI (Vazyme Bio, Nanjing). All cells were analyzed on a BD FACSCalibur Flow Cytometer (BD Biosciences, San Jose, CA) and 10,000 cells were collected with the Flow Cytometer. Cell cycle and apoptosis were analyzed by FlowJo 7.6 (Stanford University, USA). The cell cycle was set at FL2-A and apoptosis was set at FL1-H and FL2-H.

### RNA isolation and cDNA synthesis

Total RNA was extracted from the RE samples using a Trizol (Takara Bio, Otsu, Japan) extraction method [18], and Real-time PCR was carried out as described by Liu et al. [19]. A NanoDrop spectrophotometer ND-1000UV-Vis (Thermo Fisher Scientific, Madison, Wisconsin, USA) was used to detect the RNA concentration and the absorption ratio (260/280 nm) between 1.80 and 2.10, reflecting high RNA purity. A 1.4% agarose-formaldehyde gel electrophoresis was used to verify the quality of RNA samples. The concentration of RNA was adjusted to 500 ng/μL and then stored at − 80 °C. Total RNA (1 μg) was reverse-transcribed using a PrimeScript RT reagent Kit (Takara Bio Inc., Kusatsu, Japan).

### Primer design and qRT-PCR

Candidate genes related to the cell cycle protein (Cyclin), cyclin-dependent protein kinases (CDKs), IGFBPs and apoptosis proteins in the RE of sheep were evaluated using qRT-PCR. All primers were designed by Primer Premier 5.0 software (Premier Biosoft International, Palo Alto, CA) and identified using the BLAST computer program (National Center for Biotechnology Information, Bethesda, MD, USA). Primers were synthesized in Invitrogen Biological Technologies (Shanghai, China). The range in efficiency for the primers was between 92.1 and 119.0%. Real-time quantitative PCR for candidate genes and *GAPDH* were carried out using the QuantStudio 5 Real-time PCR Instrument (Applied Biosystems, Foster, California, USA), with fluorescence detection of SYBR green dye. Amplification conditions were set as follows: 95 °C for 30 s, and 40 cycles of 95 °C for 5 s and 60 °C for 30 s, followed by a dissociation curve step (95 °C for 15 s, 60 °C for 1 min, and 95 °C for 15 s). PCR products were sequenced to verify their identity (Invitrogen Biological Technologies, Shanghai, China) and all amplicons were verified as 100% homologous to their target sequence. The primers and amplicon sizes of all genes are presented in Additional file 2: Table S1). All measurements were performed in triplicate. Reverse-transcription-negative blanks of each sample served as the negative controls. Gene expression was normalized to *GAPDH* mRNA levels, and the data were analyzed according to the 2^-△△Ct^ method.

### Western blot analysis

Total protein was extracted from rumen epithelium tissue with RIPA Lysis Buffer (Cat. P0013B, Beyotime Institute of Biotechnology, Shanghai, China). The protein concentration was then detected using an enhanced BCA protein assay kit (Beyotime Institute of Biotechnology, Shanghai, China). Each protein sample (100 mg) was mixed with loading buffer and denatured at 100 °C for 5 min. Sample proteins (50 μg) and a dual color prestained broad molecular weight protein marker (Thermo Fisher Scientific, MA, USA) were separated with 10% sodium dodecyl sulfate-polyacrylamide gel electrophoresis (SDS-PAGE). The separated proteins were then transferred onto a polyvinylidene fluoride (PVDF) membrane (Merck Millipore Corporation, USA). The membrane was saturated with 5% (wt/vol) nonfat milk powder (Yili, China), prepared in Tris-buffered saline containing 0.1% Tween 20 (TBST), for 2 h at room temperature and then incubated with primary antibodies overnight at 4 °C. The primary antibodies employed were rb-anti-Cyclin D1 (Abcam, ab134175, 1:50,000 dilution), rb-anti-Cyclin A2 (Abcam, ab181591, 1:2,000 dilution), rb-anti-Cyclin E1 (Abcam, ab33911, 1:2,000 dilution), rb-anti-CDK 2 (Abcam, ab32147, 1:10,000 dilution), rb-anti-CDK 4 (Abcam, ab199728, 1:2,000 dilution), and rb-anti-CDK 6 (Abcam, ab124821, 1:50,000 dilution). After several washes with TBST, membranes were incubated in goat-anti-rabbit lgG HRP-conjugated secondary antibody (Fcmacs Biotechnology, Nanjing, China) with a 1:5,000 dilution. Then, membranes were incubated in HRP-conjugated mouse monoclonal GAPDH antibody (Santa Cruz, sc-32233, California, USA) with a 1:200 dilution to normalize the results. The signals were detected with a Immobilon Western chemiluminescent HRP substrate (Merck Millipore Corporation, USA), according to the manufacturer’s instructions, and visualized by luminescence imaging (LAS-4000, Fujifilm, Tokyo, Japan). The density of the blotting bands was then analyzed using Image J software (National Institutes of Health, Bethesda, MD).

### Statistical analysis

Comparisons between the groups were carried out with one-way ANOVA, followed by Tukey’s multiple comparison test in SPSS software packages (SPSS version 16.0.1 for Windows; SPSS Inc., Chicago, IL, USA). Linear, quadratic or cubic polynomial contrasts were performed to test for a trend in the treatment means. Differences were considered significant at *P* < 0.05, and trends were discussed at 0.05 < *P* < 0.10.

## Results

### Animals

**Fig. 1:**
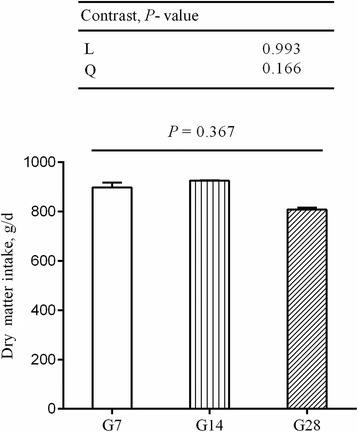
DMI during a high-grain (HG) feeding period (G7–28). Error bars indicate SEM. Values are mean ± SEM, *n* = 5. Sheep assigned to G7 (*n* = 5), G14 (*n* = 5) and G28 (*n* = 5) received a high-grain diet for 7, 14 and 28 d, respectively. Contrast patterns: L = linear, Q = quadratic

All animals were clinically healthy for the entire duration of the feeding experiments. During the hay-fed period, hay intake was approximately 1,000 g per animal per day. During the HG feeding period, there was no significant variation in DMI between the different treatment groups (*P* = 0.379) (Fig. 1). The final body weight of sheep increased linearly (*P* = 0.003) with the number of days fed an HG diet and was greater (*P* = 0.024) in the G28 group compared with the CON group (Table 2).

**Table 2:** Final body weight, ruminal pH, concentrations of ruminal volatile fatty acid (VFA) in ruminal fluid and level of IGF-1 in blood of hay-fed (CON) and high-grain-fed sheep (G7–28)

Item	Treatment				SEM	Contrast, *P*			
	CON	G7	G14	G28		Treatment	Linear	Quadratic	Cubic
Final body weight, kg	25.73^b^	26.24^ab^	28.40^ab^	29.23^a^	0.51	0.024	0.003	0.845	0.47
Rumen parameters									
Ruminal pH	6.81^a^	5.99^b^	5.94^b^	5.84^b^	0.09	< 0.001	< 0.001	< 0.001	0.106
Acetate, mmol/L	66.26	69.33	70.36	74.53	1.74	0.436	0.117	0.877	0.744
Propionate, mmol/L	18.45	19.33	19.88	23.85	1.08	0.315	0.095	0.474	0.695
Isobutyrate, mmol/L	0.61	0.81	0.68	0.78	0.04	0.363	0.343	0.563	0.165
Butyrate, mmol/L	5.74^b^	16.03^a^	20.17^a^	18.78^a^	1.41	< 0.001	< 0.001	< 0.001	0.906
Isovalerate, mmol/L	0.53	0.87	0.64	0.87	0.07	0.198	0.185	0.664	0.096
Valerate, mmol/L	0.67^b^	1.36^a^	1.29^a^	1.32^a^	0.09	0.008	0.008	0.029	0.188
Total VFA, mnol/L	92.26^b^	107.73^ab^	113.02^a^	120.13^a^	3.24	0.006	0.001	0.402	0.588
Blood parameter									
IGF-1 in plasma, ng/mL	195.88^b^	255.74^ab^	329.70^a^	293.35^a^	15.15	0.004	0.002	0.043	0.221

Sheep assigned to CON (*n* = 5), G7 (*n* = 5), G14 (*n* = 5) and G28 (*n* = 5) received a high-grain diet for 0, 7, 14 and 28 d, respectively

*SEM* standard error of mean

^a, b^Means within a row with different superscripts differ (*P* < 0.05)

### Rumen fermentation and plasma IGF-1

The rumen pH, concentrations of ruminal VFA in ruminal fluid and the level of IGF-1 in the blood are summarized in Table 2. Feeding the HG diet linearly decreased (*P* < 0.001) the ruminal pH. Total rumen VFA concentration increased (linear, *P* = 0.001) with HG diet feeding and was greater in the G14 and G28 groups than in the CON group (*P* = 0.006). Furthermore, the butyrate concentration increased linearly (*P* < 0.001) with the number of days fed the HG diet. The valerate concentration increased quadratically (*P* = 0.029) and was greater in the G7, G14 and G28 groups (*P* = 0.008) than in the CON group. No significant changes were observed in the concentrations of acetate (*P* = 0.436), propionate (*P* = 0.315), isobutyrate (*P* = 0.363) and isovalerate (*P* = 0.198) among the four groups. Feeding the grain diet quadratically increased (*P* = 0.043) the plasma IGF-1 concentration, and the level of IGF-1 in plasma was greater in the G14 and G28 groups (*P* = 0.004) than in the CON group.

### Rumen papillae density, dimensions and surface area

To evaluate whether dietary adaptation affected rumen papillae density, papillae dimensions and surface, 1-cm^2^ pieces of RE samples were collected and measured immediately after the animals’ slaughter. The results are shown in Table 3. No significant differences were observed (*P* = 0.865) in the papillae density of the ventral sac among the four groups. However, there was a quadratic increase (*P* = 0.004) in papillae length from 3.66 for CON group to 4.76 mm for G28 group, with the greatest length being 5.32 mm for the G14 group. Papillae width increased cubically (*P* = 0.015), and was greater in the G28 group than in the CON and G14 groups (*P* = 0.001). Furthermore, the two-dimensional surface area of rumen papillae was calculated, and the data showed that it increased linearly (*P* = 0.003) with the number of days fed an HG diet, and it was greater (*P* = 0.010) in the G14 and G28 groups compared with the CON and G7 groups.

**Table 3:** Rumen papillae density, dimensions and surface area of hay-fed (CON) and high-grain-fed sheep (G7–28)

Item	Treatments				SEM	Contrast, *P*			
	CON	G7	G14	G28		Treatment	Linear	Quadratic	Cubic
Length, mm	3.66^c^	3.72^c^	5.32^a^	4.76^b^	0.15	< 0.001	< 0.001	0.004	< 0.001
Width, mm	1.90^b^	1.98^ab^	1.89^b^	2.13^a^	0.06	0.001	0.004	0.093	0.015
Surface, mm/cm^2^	935.00^b^	1077.83^ab^	1407.49^a^	1337.98^a^	136.58	0.010	0.003	0.288	0.194
Density, no./cm^2^	70	76	74	70	10	0.865	0.964	0.420	0.843

Sheep assigned to CON (*n* = 5), G7 (*n* = 5), G14 (*n* = 5) and G28 (*n* = 5) received a high-grain diet for 0, 7, 14 and 28 d, respectively

*SEM* standard error of mean

^a-c^ Means within a row with different superscripts differ (*P* < 0.05)

### Rumen epithelium morphology

The representative rumen papillae cross sections are shown in Additional file 1: Figure S1. The changes in the rumen papillae morphology of the RE in CON, G7, G14 and G28 groups are described in Table 4. The number of cell layers forming SC increased cubically (*P* < 0.001) with HG diet feeding, and was greater in the G7 group than in the CON group (*P* = 0.004). The thickness of SC increased cubically (*P* < 0.001) and was greater (*P* < 0.001) in G7 and G28 groups than in CON and G14 groups. The thickness of the sum of the SG and SS decreased cubically (*P* < 0.001), and was lower in G7 and G14 groups relative to CON and G28 groups (*P* < 0.001). Feeding the grain diet cubically increased (*P* = 0.028) the thickness of the SB, and the SB thickness was greater (*P* < 0.001) in G28 group than in CON, G7 and G14 groups.

**Table 4:** Rumen papillae morphology of hay-fed (CON) and high-grain-fed sheep (G7–28)

Item	Treatments				SEM	Contrast, *P*			
	CON	G7	G14	G28		Treatment	Linear	Quadratic	Cubic
Thickness of stratum corneum, μm	21.31^b^	27.12^a^	20.26^b^	26.18^a^	0.78	< 0.001	< 0.001	< 0.001	< 0.001
Cell layers of stratum corneum, number of cell layers	2.86^b^	3.13^a^	2.98^ab^	2.99^ab^	0.04	0.004	< 0.001	0.006	< 0.001
Thickness of stratum granulosum/spinosum, μm	87.88^a^	69.70^b^	69.28^b^	88.16^a^	1.92	< 0.001	0.006	0.410	< 0.001
Thickness of stratum basale, μm	11.50^b^	10.51^b^	10.63^b^	13.66^a^	0.25	< 0.001	0.002	0.016	0.028

Sheep assigned to CON (*n* = 5), G7 (*n* = 5), G14 (*n* = 5) and G28 (*n* = 5) received a high-grain diet for 0, 7, 14 and 28 d, respectively

*SEM* standard error of mean

^a, b^ Means within a row with different superscripts differ (*P* < 0.05)

### Analyses of cell cycles and apoptosis

The representative cell cycles of RE are shown in Additional file 3: Figure S2 and the details are shown in Table 5. The proportion of cells in the G_0_/G_1_-phase was lower (*P* < 0.001) in the G14 and G28 groups than in the CON and G7 groups (linear *P* < 0.001). However, the proportion of cells in S-phase increased linearly (*P* = 0.006), and was greater (*P* = 0.007) in G28 group than in CON, G7 and G14 groups, while the cell number percentage of the G_2_/M-phase increased (*P* = 0.004) cubically, and was greater in G14 and G28 groups than in CON and G7 groups (*P* < 0.001). The changes in cell apoptosis in the RE (Additional file 4: Figure S3) were also evaluated. The results showed that the proportion of apoptosis cells in G7, G14 and G28 groups was reduced compared with CON group (quadratic, *P* < 0.001). However, the proportion of apoptosis cells increased from 19.03% for G7 group to 28.22% for G14 group, with an increase to 38.33% for G28 group (*P* < 0.05).

**Table 5:** Cell cycle progression of ruminal epithelial cells of hay-fed (CON) and high-grain-fed sheep (G7–28)

Item^1^	Treatments				SEM	Contrast, *P*			
	CON	G7	G14	G28		Treatment	Linear	Quadratic	Cubic
G_0_/G_1_, %	91.77^a^	89.96^a^	85.86^b^	84.73^b^	0.76	< 0.001	< 0.001	0.754	0.238
S, %	2.78^b^	2.56^b^	3.05^b^	5.11^a^	0.33	0.007	0.006	0.058	0.691
G_2_/M, %	4.24^b^	5.62^b^	9.70^a^	9.36^a^	0.56	< 0.001	< 0.001	0.058	0.004
Apoptosis cells, %	62.72^a^	19.03^d^	28.22^c^	38.33^b^	3.82	< 0.001	< 0.001	< 0.001	< 0.001

Sheep assigned to CON (*n* = 5), G7 (*n* = 5), G14 (*n* = 5) and G28 (*n* = 5) received a high-grain diet for 0, 7, 14 and 28 d, respectively

*SEM* standard error of mean

^a-d^ Means within a row with different superscripts differ (*P* < 0.05)

^1^ G_0_/G_1_ = the proportion of cells in the gap G_0_ and G_1_ phases (the percentage is based on 10,000 cells); S = the proportion of cells in the synthesis (S) phase, based on the fact that the single-cell DNA level is greater than the DNA content of resting diploid cells (the percentage is based on 10,000 cells measured); G_2_/M = the proportion of mitotic cells (the percentage is based on 10,000 cells). Apoptosis cells = the proportion of cells in the apoptosis phase (the percentage is based on 10,000 cells measured)

### Gene and protein expression related to cell-cycle-regulation

**Fig. 2:**
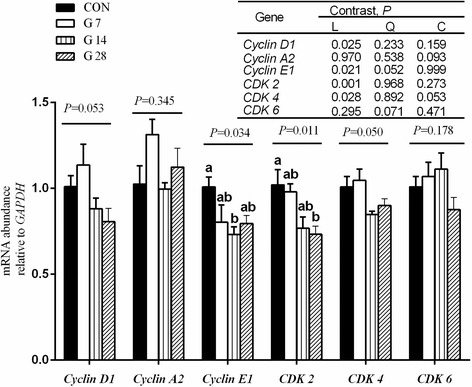
Expression of genes related to cell cycle regulation in the ruminal epithelium of hay-fed (CON) and high-grain-fed sheep (G7–28). Values are mean ± SEM, *n* = 5. ^a, b^ Values within a column differ if they do not share a common superscript (*P* < 0.05). Sheep assigned to CON (*n* = 5), G7 (*n* = 5), G14 (*n* = 5) and G28 (*n* = 5) received a high-grain diet for 0, 7, 14 and 28 d, respectively. Contrast patterns: L = linear, Q = quadratic, C = cubic

The expression of genes related to cell-cycle-regulating proteins (*Cyclin D1*, *Cyclin A2*, *Cyclin E1*, *CDK-2*, *CDK-4* and *CDK-6*) are shown in Fig. 2. The mRNA expression of *Cyclin E1* linearly decreased (*P* = 0.021) from 1.00 for CON group to 0.73 for G14 group, and *CDK-2* gene expression decreased from 1.02 for CON group to 0.73 for G28 group (linear, *P* = 0.001). No significant changes (*P* ≥ 0.05) were observed in the mRNA expression of *Cyclin D1*, *Cyclin A2*, *CDK-4* and *CDK-6* among the four groups.

**Fig. 3:**
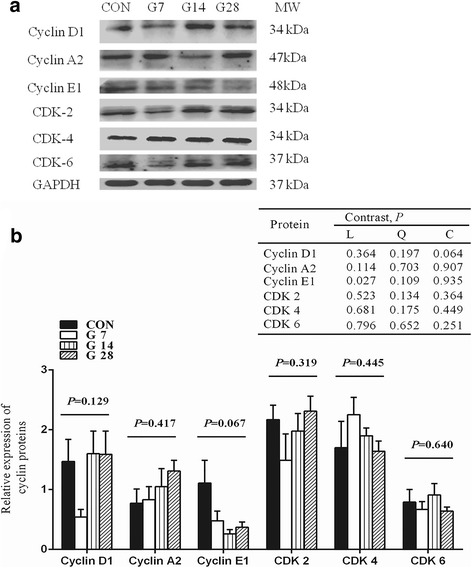
Expression of proteins related to cell cycle regulators in rumen epithelial tissue of hay-fed (CON) and high-grain-fed sheep (G7–28). Protein extracts of rumen epithelium samples were prepared and immunoblotted with specific antibodies (**a**). Intensities of Cyclin D1, Cyclin A2, Cyclin E1, CDK-2, CDK-4 and CDK-6 were normalized to corresponding GAPDH levels (**b**) (means ± SE; *n* = 5). Sheep assigned to CON(*n* = 5), G7(*n* = 5), G14(*n* = 5) and G28(*n* = 5) received a high-grain diet for 0, 7, 14 and 28 d, respectively. Contrast patterns: L = linear, Q = quadratic, C = cubic

The protein expressions of Cyclin A2, Cyclin D1, Cyclin E1, CDK 2, CDK 4 and CDK 6 were assessed by Western blotting (Fig. 3). The results showed that the protein expression of Cyclin E1 decreased linearly (*P* = 0.027) from 1.11 for CON group to 0.37 for G28 group, with a minimum expression at 0.26 for G14 group. No significant differences were observed for the expression of Cyclin D1 (*P* = 0.129), Cyclin A2 (*P* = 0.417), CDK 2 (*P* = 0.319), CDK 4 (*P* = 0.445) or CDK 6 (*P* = 0.640) among the four groups.

### Gene expression related to the IGF-1 pathway and cell apoptosis

**Fig. 4:**
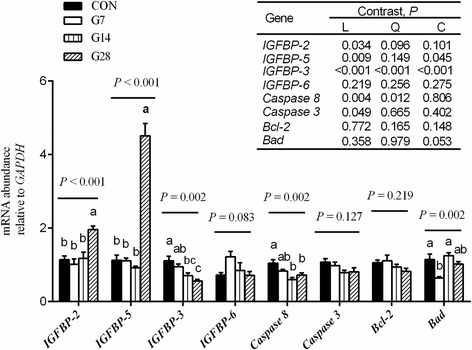
Expression of genes that regulated the IGF-1 pathway and cell apoptosis in the ruminal epithelium of hay-fed (CON) and high-grain-fed sheep (G7–28). Values are mean ± SEM, *n* = 5. ^a-c^ Values within a column differ if they do not share a common superscript (*P* < 0.05). Sheep assigned to CON(*n* = 5), G7(*n* = 5), G14(*n* = 5) and G28(*n* = 5) received a high-grain diet for 0, 7, 14 and 28 d, respectively. Contrast patterns: L = linear, Q = quadratic, C = cubic

The expression of genes that regulate the IGF-1 pathway, namely *IGFBP 2*, *IGFBP 3*, *IGFBP 5* and *IGFBP 6,* and cell apoptosis, namely *Caspase 3*, *Caspase 8*, *Bcl-2* and *Bad*, were evaluated by real-time quantitative PCR (Fig. 4). The mRNA expression of *IGFBP 2* (linear, *P* = 0.034) and *IGFBP 5* (linear, *P* < 0.009) increased up to the maximal level after 28 d of HG diet feeding, while *IGFBP 3* mRNA expression decreased linearly (*P* < 0.001) from 1.10 for CON group to 0.56 for G28 group, and *IGFBP 6* expression tended to increase (*P* = 0.083) from CON group to the G7 group.

The mRNA expression of *Caspase 8* decreased (quadratic, *P* = 0.012) from 1.04 for CON group to 0.60 for G14 group and 0.72 for G28 group, but there was no difference (*P* > 0.0*5*) among G7, G14 and G28 groups. *Bad* mRNA expression tended to decrease (cubic, *P* = 0.053) from 1.14 for CON group to 0.64 for G7 group, with an increase (*P* < 0.05) to 1.24 for G14 group compared with G7 group. However, the expressions of *Caspase 3* (*P* = 0.127) and *Bcl-2* (*P* = 0.219) showed no variation among the four groups.

## Discussion

In the present study, our data revealed that HG feeding significantly increased the concentrations of total VFA and decreased the ruminal pH, which were in line with some previous studies [19–21]. In response to increased VFA production and decreased ruminal pH, our data revealed that the RE responded to these challenges in a coordinated manner. The present study showed that the length, width and surface area of the rumen papillae were significantly enlarged with increasing time of concentrate feeding in the diet, which was consistent with previous studies on goats [22] and cattle [23–25]. An enlargement of the RE surface area could enhance transport rates and increase the activity of the transport proteins involved in VFA absorption and regulation of intracellular pH [26, 27], further stimulating VFA absorption and pH value recovery in the rumen [13, 28, 29]. In addition, the present study showed that the duration of time required for a significant increase in ruminal papillae surface area was at least 2 wk after HG feeding, which was consistent with the report by Liebich et al. [30], who showed that the size of papillae increased at the 2-wk mark after switching from a dry period to a lactation diet.

The increase in ruminal VFA production and acidic load associated with HG feeding necessitates an adaptation of RE structure and function [8, 31]. The present data indicated that, after 7 d of HG diet feeding, the thickness and layers of SC increased cubically, and the increase in the thickness of the SB occurred within 28 d of the onset of HG feeding. These results were similar to other reports [32, 33], which demonstrated that the cell layers and thickness of the SC increased with increasing levels of concentrate feeds in the diet.

Enlarged rumen papillae size caused by HG feeding may be the result of an accelerated cell cycle progression [15, 20]. Previous studies revealed that the animal cell cycle consists of five distinct but sequential phases named the G_0_, G_1_, S, G_2_ and M phase, respectively [15, 20, 34]. The cells in the G_0_ phase have normal function (but not mitotic activity) and become quiescent; the G_1_ phase is the phase of cell growth; the S phase is the phase of DNA synthesis; the G_2_ phase includes preparations for cell division; and the M phase is the cell division phase [34]. In the present study, HG feeding resulted in a decrease in the proportion of RE cells in the G_0_/G_1_-phase and an increase in the percentage of RE cells in the S-phase and G_2_/M-phases. This indicated that the time duration of the G_0_/G_1_-phase was shortened, and then, cell cycle progression was accelerated. These changes may lead to an increase in rumen papillae size, which further enhances the absorption ability of the RE [10, 15, 20]. In addition, the present study showed that significant changes in the proportion of the basal layer cells of the RE in the G_0_/G_1_-phase and G_2_/M-phase occurred at d14 after HG diet feeding, indicating that the acceleration of cell division in the RE occurred after 2 wk of HG diet feeding.

Cyclins and cyclin-dependent kinases (*CDKs*) are two main protein families that control cell cycle progression [35, 36]. The G_1_-phase cell cyclin protein contains Cyclin D and Cyclin E [34]. The Cyclin D1 combines with CDK 4 and CDK 6, forming complexes to enter into the G_1_-phase [37]. The Cyclin E-*CDK 2* complex promotes progression from G_1_ into the S phase [38]. Any changes in the duration of one or more phases of the cell cycle will affect cell cycle progression and cell growth [34]. A previous study showed that the Cyclin E-*CDK 2* complex accumulated at the G_1_/S-phase boundary and is degraded as cells progress through the S phase [39]. In the present study, compared with the CON group, the *Cyclin E 1* and *CDK 2* mRNA expression was lower at 14 and 28 d, respectively, after the onset of HG feeding. Similar changes in the protein expression of Cyclin E 1 decreased with a minimum expression at 14 d after HG feeding. This indicated that Cycline E1 and CDK 2 may be degraded as cells progress from S into the G_2_/M phase [40]. However, no similar changes were observed in the protein expression of CDK 2. As shown in other studies [41, 42], changes in protein levels did not automatically correlate well to mRNA levels, implying that posttranscriptional events may contribute to the formation of such discrepancies.

The results of this study showed that plasma IGF-1 concentration increased linearly with the number of days fed an HG diet, and this finding is consistent with the previous report by Shen et al. [22], who found that plasma IGF-1 concentration increased significantly at 14 d (191 ± 21 μg/L) and 21 d (302 ± 36 μg/L) after feeding a high-energy diet in goats aged 4 months. Some previous studies also revealed that energy intake had marked effects on the plasma IGF-1 concentration in sheep [20, 22]. Thus, in the current study, the higher plasma IGI-1 level in the HG group might be explained by the high-energy intake in sheep fed an HG diet. Consistent with some previous reports that found HG diet feeding increased the final body weight of sheep [43, 44], the current study also revealed that the BW at slaughter increased linearly with the days fed an HG diet.

It has been well established that HG feeding or the intraruminal infusion of VFA can stimulate the proliferation of RE [4, 45], and the growth promoters such as VFA and IGF may be involved in the regulation of proliferation [8, 22]. Shen et al. showed that increasing dietary energy could improve plasma IGF-1 concentrations in goats and further stimulate rumen papillae growth and enhance the functional adaptation of ruminal Na^+^ absorption. [22, 46]. Zitnan et al. reported that plasma IGF-1 concentrations had been positively associated with ruminal papillae proliferation in calves [47]. As a growth factor, IGF-1 was believed to play a pivotal role in RE growth [20], as it induced RE cell proliferation in vitro [11, 22], thereby initiating a signal transduction cascade that activates genes responsible for proliferation and apoptosis [48].

IGF-1 induces cellular response by regulating IGF-binding proteins (IGFBPs) [49]. It has been well characterized that IGFBP 2, IGFBP 3 and IGFBP 5 were thought to modulate IGF-1 cellular events [50, 51]. Of these IGFBPs, IGFBP 5, which is known to potentiate IGF-1 effects, may encourage proliferation in the RE when upregulated, whereas IGFBP 3 modulates IGF-1 cellular events in an opposing fashion to IGFBP 5. In the present study, our data revealed that HG feeding upregulated the mRNA expression of *IGFBP 2* and *IGFBP 5*, and downregulated the mRNA expression of *IGFBP 3*, which was in line with the report by Steele et al. [1], who showed that the mRNA expression of *IGFBP 3* was down-regulated, and *IGFBP 5* was up-regulated in the RE of nonlactating cattle after transfer to a high-grain diet [5]. Thus, it is reasonable to suppose that IGF-1 may trigger the proliferation of the RE and block apoptosis to increase growth after 2 wk of HG feeding.

Butyrate has commonly been regarded as one of the most potent stimulators of epithelial proliferation in the digestive tract [52–54]. Previous studies revealed that the intraruminal infusion of n-butyric acid stimulated ruminal papillae growth in vivo [55, 56]. In contrast to in vivo studies, butyrate led to an inhibitory effect on RE proliferation in vitro [11, 57]. These data showed that butyrate did not accelerate epithelial proliferation directly, but did so through other mechanisms. Mentschel et al. reported that intraruminal butyrate infusions induced papillae growth by decreasing apoptosis in the ruminal epithelium [12], and the mechanism underlying these alterations may be related to the downregulation of the *IGFBP 3* expression induced by butyrate [58, 59]. Interestingly, the present study showed that the proportion of apoptosis cells was decreased and the mRNA expression of apoptosis genes *Caspase 8* and *Bad* was downregulated by HG feeding, indicating that the RE proliferation might be partly caused by the slow apoptotic rate of the RE. In addition, the present study found that the concentration of butyrate increased after 1 wk of HG feeding. Thus, it is reasonable to suppose that butyrate may trigger the downregulation of *IGFBP 3* in the RE, thus blocking apoptosis and increasing growth. Nevertheless, the underlying mechanism of the ruminal epithelial apoptosis suppressed by butyrate still needs to be further evaluated.

## Conclusions

In summary, the present study revealed that HG feeding resulted in changes in the mRNA expression of *Cyclin E1* and *CDK 2*, and the protein expression tendency of Cyclin E1. It also found that molecular markers (*IGFBP 2*, *IGFBP 5*, *Caspase 8* and *Bad*) may play a role in signaling the adaptive response of the RE to HG diet feeding in sheep. These findings provide new insights into the sequential changes of rumen papillae size, cell cycle regulation and the molecular adaptive mechanisms of the RE during HG diet feeding in sheep, suggesting that the morphology adaptation and cell-cycle-regulating adaptation of the RE to HG feeding are procedural adaptations. These findings enhanced our understandingof the molecular adaptive mechanisms of the RE and the functional changes in the RE in response to HG diet feeding, which may be helpful in developing technologies or feeding strategies to increase productivity in feedlot sheep and cattle.

## Additional files

Additional file 1: **Figure S1.** Rumen papillae histology (scale bar = 100 μm) of hay-fed (A: CON) and high-grain diet-fed sheep (B: G7; C: G14; D: G28). (SC, stratum corneum; SG, stratum granulosum; SS, stratum spinosum; SB, stratum basale). Sheep assigned to CON (*n* = 5), G7 (*n* = 5), G14 (*n* = 5) and G28 (*n* = 5) received a high-grain diet for 0, 7, 14 and 28 d, respectively. The stratum corneum (SC) was the outermost cell layer, which was heavily stained and the stratum granulosum (SG) was defined as the layer of long axes cells which lay perpendicular to the stratum spinosum (SS) and basale (SB). SS and SB were cells nested between the lamina propria and SG. (TIFF 6443 kb)

Additional file 2: **Table S1.** Primers for quantitative real time PCR. (DOCX 18 kb)

Additional file 3: **Figure S2.** Cell cycle distribution in the ruminal epithelium of sheep fed purely hay (A: CON) and a high-grain diet for 7 (B: G7), 14 (C: G14) and 28 d (D: G28). (TIFF 468 kb)

Additional file 4: **Figure S3.** Cell apoptosis distribution in the ruminal epithelium of sheep fed purely hay (A: CON) and a high-grain diet for 7 (B: G7), 14 (C: G14) and 28 d (D: G28). Q4: Nonapoptotic, live cells do not bind Annexin V-FITC and exclude PI(Propidium lodide); Q3: Early apoptotic cells bind Annexin V-FITC and exclude PI; Q2: Late apoptotic cells bind Annexin V-FITC and also PI. Apoptosis cells = Q2 (Late apoptotic cells) + Q3 (Early apoptotic cells). (TIFF 1543 kb)

## Abbreviations

BW: Body weight
CDKs: Cyclin-dependent protein kinases
CON: Control
Cyclin: Cell cycle protein
DMI: Dry matter intake
HG: High-grain
IGF-1: Insuline like growth factor 1
IGFBPs: IGF-binding proteins
RE: Ruminal epithelium
SB: Stratum basale
SC: Stratum corneum
SG: Stratum granulosum
SS: Stratum spinosum
VFA: Volatile fatty acid
