# Supplementary figures and images for: Morphological adaptation of sheep’s rumen epithelium to high-grain diet entails alteration in the expression of genes involved in cell cycle regulation, cell proliferation and apoptosis
Source: J Anim Sci Biotechnol. 2018 Apr 16;9:32. doi: 10.1186/s40104-018-0247-z (PMC5901869; doi:10.1186/s40104-018-0247-z)

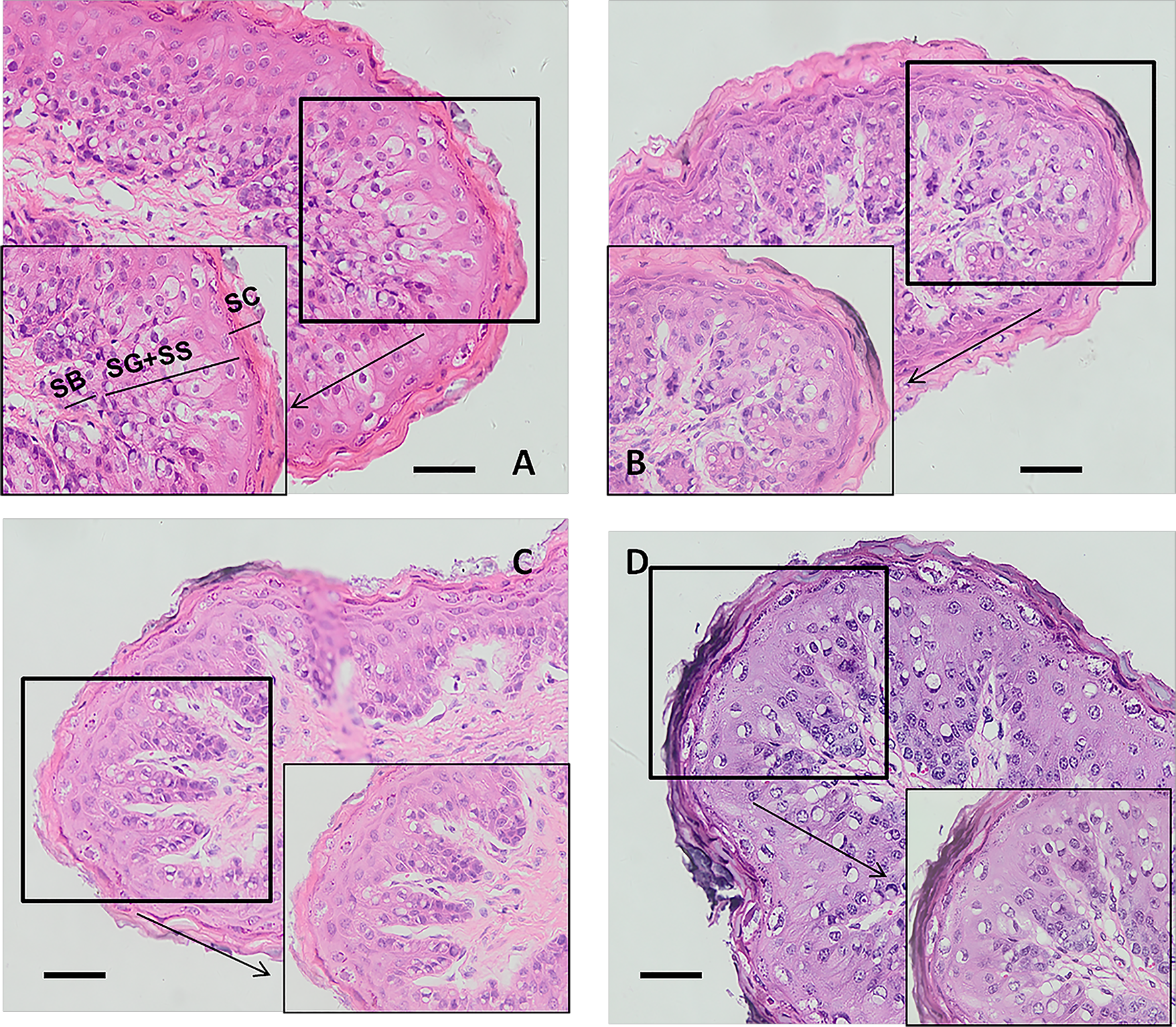

Supplement: Supplementary file 1 — Figure S1. Rumen papillae histology (scale bar = 100 μm) of hay-fed (A: CON) and high-grain diet-fed sheep (B: G7; C: G14; D: G28). (SC, stratum corneum; SG, stratum granulosum; SS, stratum spinosum; SB, stratum basale). Sheep assigned to CON (n = 5), G7 (n = 5), G14 (n = 5) and G28 (n = 5) received a high-grain diet for 0, 7, 14 and 28 d, respectively. The stratum corneum (SC) was the outermost cell layer, which was heavily stained and the stratum granulosum (SG) was defined as the layer of long axes cells which lay perpendicular to the stratum spinosum (SS) and basale (SB). SS and SB were cells nested between the lamina propria and SG. (TIFF 6443 kb) [file 40104_2018_247_MOESM1_ESM.tif]

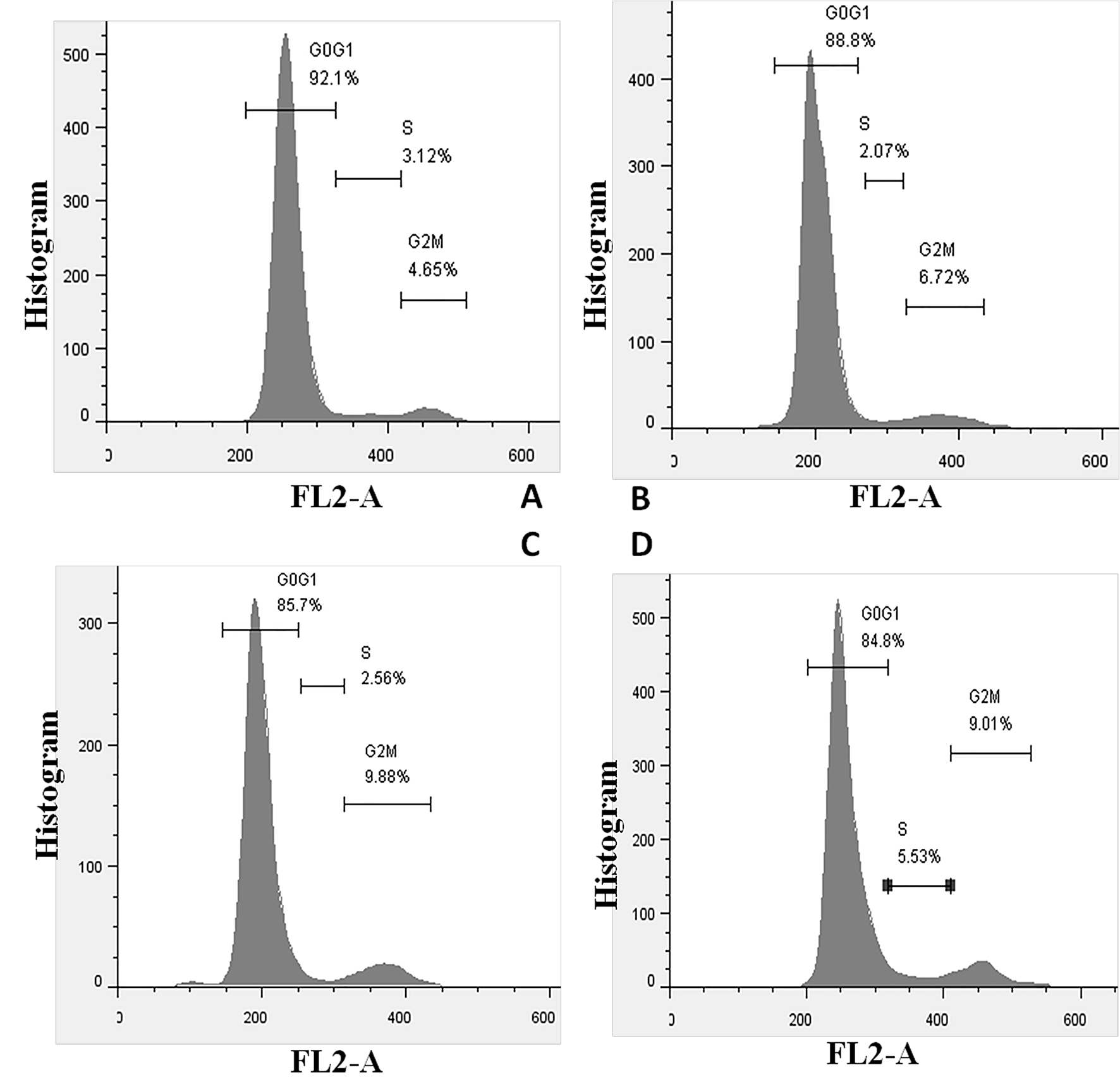

Supplement: Supplementary file 3 — Figure S2. Cell cycle distribution in the ruminal epithelium of sheep fed purely hay (A: CON) and a high-grain diet for 7 (B: G7), 14 (C: G14) and 28 d (D: G28). (TIFF 468 kb) [file 40104_2018_247_MOESM3_ESM.tif]

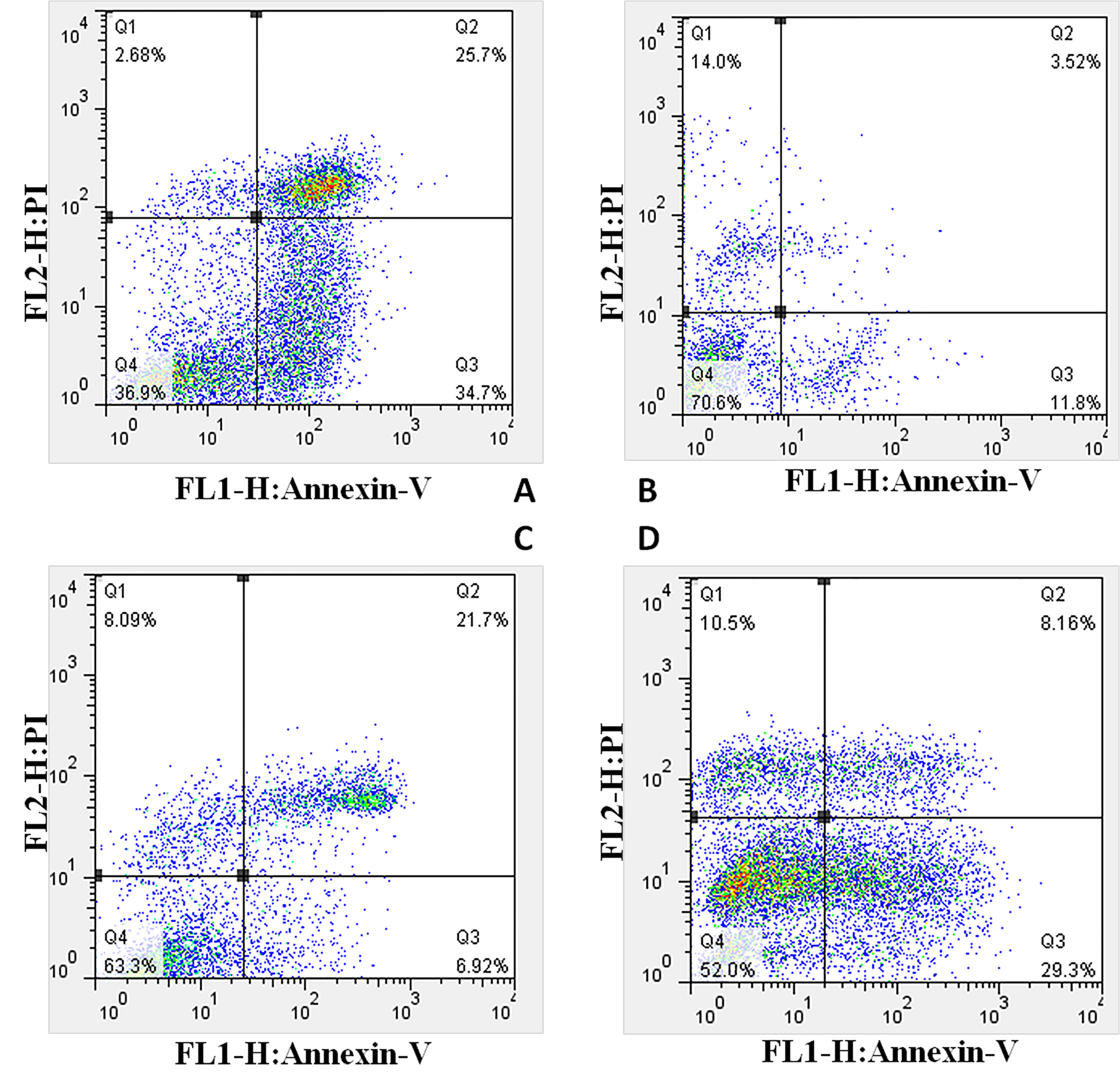

Supplement: Supplementary file 4 — Figure S3. Cell apoptosis distribution in the ruminal epithelium of sheep fed purely hay (A: CON) and a high-grain diet for 7 (B: G7), 14 (C: G14) and 28 d (D: G28). Q4: Nonapoptotic, live cells do not bind Annexin V-FITC and exclude PI(Propidium lodide); Q3: Early apoptotic cells bind Annexin V-FITC and exclude PI; Q2: Late apoptotic cells bind Annexin V-FITC and also PI. Apoptosis cells = Q2 (Late apoptotic cells) + Q3 (Early apoptotic cells). (TIFF 1543 kb) [file 40104_2018_247_MOESM4_ESM.tif]
