# Supplementary material for: Morphological adaptation of sheep’s rumen epithelium to high-grain diet entails alteration in the expression of genes involved in cell cycle regulation, cell proliferation and apoptosis
Source: J Anim Sci Biotechnol. 2018 Apr 16;9:32. doi: 10.1186/s40104-018-0247-z (PMC5901869; doi:10.1186/s40104-018-0247-z)
Supplement: Supplementary file 2 — Table S1. Primers for quantitative real time PCR. (DOCX 18 kb) [file 40104_2018_247_MOESM2_ESM.docx]

**Table S1.** Primers for quantitative real time PCR.

| Gene Name^1^ | Gene ID | Primer sequence (5’→3’)^2^ | Amplicon Size, bp |
| --- | --- | --- | --- |
| *Cyclin A2* | NC_019478.2 | F: CTCTCCTATCACCGCCTGAC  R: CTTTGGGGTCCAAGTTCTGC | 142 |
| *Cyclin D1* | XM_015102997.1 | F: CGATAAGCCAGCTAACGGGG  R: CAGGCTGCCTCCGTCTG | 103 |
| *Cyclin E1* | XM_015100542.1 | F: TGGCACCGATGTCTCTGTTC  R: CCACACTGGCTTCTCACAGT | 114 |
| *CDK2* | NC_019460.2 | F: CCTAGCTTTCTGCCACTCTCAT  R: TCACCACCTCGTGGGTATAAGT | 155 |
| *CDK4* | NM_001127269 | F: GACCAAGACCTCAGGACGTATC  R: CACCACTTGTCACCAGAATGTT | 114 |
| *CDK6* | NC_019461.2 | F: GATGGCTCTTACCTCAGTGGTT  R: GGGTAGGGCAACATCTCTAGG | 94 |
| *GAPDH* | HM043737.1 | F: ACAGTCAAGGCAGAGAACGG  R: CCAGCATCACCCCACTTGAT | 98 |
| *IGFBP-2* | NM_001009436.1 | F: GTCCTGGAACGGATCTCCAC  R: GAGGTTGTACAGGCCATGCT | 108 |
| *IGFBP-3* | NM_001159276.1 | F: AAATGGAGGACACACTGAACG  R: TTATCCACACACCAGCAGAAAC | 152 |
| *IGFBP-5* | EU727460.1 | F: TGAAGGCTGAGGCTGTGAAG  R: GGCCCCTGCTCAGATTCC | 133 |
| *IGFBP-6* | EU862545.1 | F: GGGTCTACACTCCCAACTGC  R: TAGGATTCTCTCCCGAGGGC | 132 |
| *Caspase 3* | XM_015104559.1 | F: CAGCTACCTCAAACACAGTTGG  R:TGATACAGTGGCATACCCACAT | 203 |
| *Caspase 8* | NC_019459.2 | F: TCCAGGATTCGCCTCTGGTA  R: CCGGCTTAGGAACTTGAGGG | 133 |
| *Bcl-2* | XM_012103831.2 | F: GTGGATGACCGAGTACCTGAAC  R: CTTCACTTATGGCCCAGATAGG | 197 |
| *Bad* | XM_004019650.3 | F: TTTCGGAAGACTGAGGTCTGAT  R: CGGCGAAGTTAGGGTTAATCTC | 185 |

^1^*CDK-2* = Cyclin-dependent Kinase 2; *CDK-4* = Cyclin-dependent Kinase 4; *CDK-6* = Cyclin-dependent Kinase 6; *IGF-1R* = Insulin-like Growth Factor 1 Receptor; *IGFBP 2* = Insulin-like Growth Factor Binding Protein 2; *IGFBP 3* = Insulin-like Growth Factor Binding Protein 3; *IGFBP 5* = Insulin-like Growth Factor Binding Protein 5; *IGFBP 6* = Insulin-like Growth Factor Binding Protein 6; *Bcl-2* = B-cell lymphoma-2; *BAD* = *Bcl-2* associated agonist of cell death.

^2^The reference sequence number is given for primers whose source is the National Center for Biotechnology Information (NCBI) GenBank database. F = Forward primer; R = Reverse primer.
